# Recurrent pneumonia in a patient with new coronavirus infection after discharge from hospital for insufficient antibody production: a case report

**DOI:** 10.1186/s12879-020-05231-z

**Published:** 2020-07-11

**Authors:** Xiaoxi Zhou, Jianfeng Zhou, Jianping Zhao

**Affiliations:** 1 Department of Hematology, Tongji Hospital, Tongji Medical College, Huazhong University of Science and Technology, 1095 Jiefang Avenue, Wuhan, 430030 Hubei China; 2 Department of Respiration, Tongji Hospital, Tongji Medical College, Huazhong University of Science and Technology, 1095 Jiefang Avenue, Wuhan, 430030 Hubei China

**Keywords:** New corona virus, Recurrence, Protective antibodies, Extend isolation time, Case report

## Abstract

**Background:**

The rapid spread of coronavirus disease 2019 (COVID-19) was declared as an emerging public health threat by the World Health Organization. As various measures have been taken successfully to combat the epidemic caused by SARS-CoV-2, a growing number of fully recovered patients have been discharged from hospitals. However, some of them have relapsed. Little is known about the causes that triggered the relapse.

**Case presentation:**

We report a case of a 40 years old man who suffered from recurrent pulmonary infection with progression of lesions on chest computed tomography (CT), elevated levels of ferritin and IL2R, reduced lymphocyte count and positive oropharyngeal swab test for SARS-CoV-2 again after 5 days discharge from hospital. The anti-SARS-CoV-2 antibody level of this patient was very low at the time of relapse, suggesting a weak humoral immune response to the virus. Total exon sequencing revealed mutations in TRNT1 gene, which may be responsible for B cell immunodeficiency. Therefore, uncleared SARS-CoV-2 at his first discharge was likely to lead to his recurrence. However, viral superinfection and non-infectious organizing pneumonia could not be completely excluded.

**Conclusion:**

COVID-19 relapse may occur in a part of discharged patients with low titers of anti-SARS-CoV-2 antibodies. These patients should be maintained in isolation for longer time even after discharge. A more sensitive method to detect SARS-CoV-2 needs to be established and serological testing for specific antibodies may be used as a reference to determine the duration of isolation.

## Background

Coronavirus disease 2019 (COVID-19), caused by infection with the severe acute respiratory syndrome coronavirus 2 (SARS-CoV-2), has now spread all over the world, since it broke out in Wuhan city, China [1, 2]. Based on the standard to discontinue isolation written in the *Guidelines for the Diagnosis and Treatment of Patients with COVID-19(version 6)-*patients can be discharged from healthcare facilities after their body temperature returned to normal for more than 3 days, with improved respiratory symptoms and clear absorption of inflammation on chest CT imaging, and 2 negative nucleic acid tests on respiratory tract pathogen over 24 h interrnal [3]. As of March 1, 2020, more than 40,000 patients in China have been released from isolation. Here, we report a case of a 40 years old man who tested positive for SARS-CoV-2 and had aggravated symptoms and worsening lesions on CT scan after leaving the hospital, which is different from previous reports [4].

## Case presentation

**Fig. 1:**
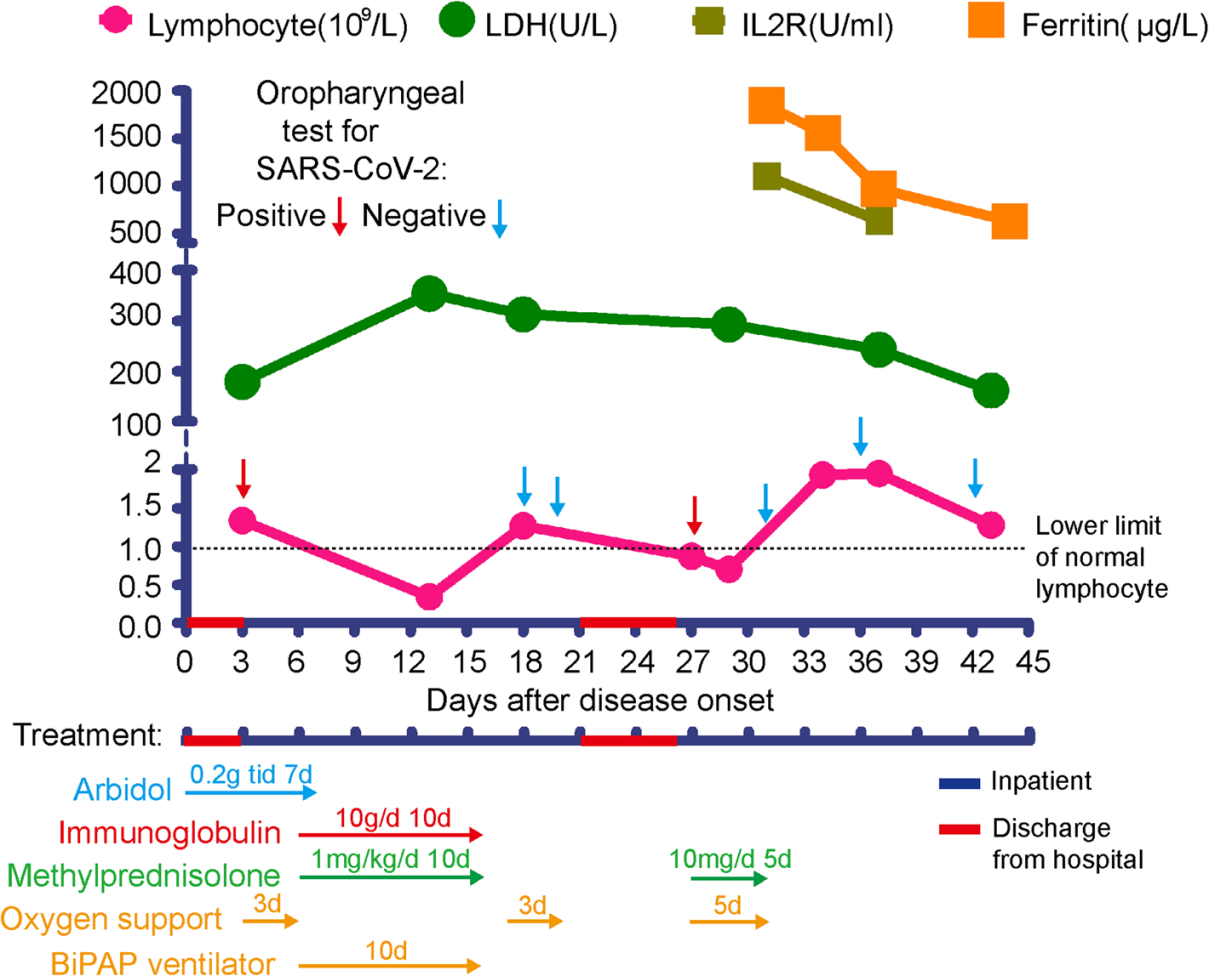
A timeline of treatment and clinical events with laboratory parameters. A timeline of treatments and clinical events and time course of LDH (green), ferritin (orange) and IL2R (green yellow) and lymphocyte number (pink) with the results of oropharyngeal swab tests for SARS-CoV-2: positive: red arrow; negative: light blue arrow

**Fig. 2:**
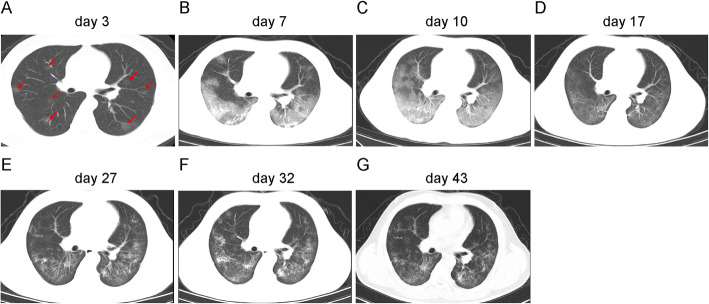
Chest CT findings. The lesions (indicated by red arrow) of CT scan detected on day 3 after symptom onset (**a**). Axial images showed bilateral ground-glass opacity and consolidation on day 9 (**b**) and the larger area of lesions were observed on day 10 (**c**). CT scan on day 17 showed significant improvement of lesions before the first discharge (**d**), and on day 27 at relapse showed consolidation with higher density (**e**). The sixth and seventh follow-up CT scans demonstrated increased lesions on day 32 (**f**) and subsequent reduction of damaged area on day 44 (**g**), respectively

A previously healthy 40-year-old male, whose mother had been diagnosed with SARS-CoV-2 infection a week ago, started to have fever without dry cough, dyspnea and diarrhea on Jan.18, 2020 (day 1). He received antivirus therapy (Arbidol) for a week because of his contact history and symptoms (Fig. 1). On Jan. 20, 2020 (day 3), the chest CT scan revealed bilateral pneumonia (Fig. 2a). He was transferred from fever clinic to isolation ward of Tongji hospital in Wuhan. On Jan. 23 (day 6), he was diagnosed with SARS-CoV-2 infection confirmed by the positive oropharyngeal swab test (detail shown in supplementary method). His inspiratory dyspnea was obvious with < 80% arterial oxygen saturation. The follow-up CT scan on Jan. 24 (day 7) and 27 (day 10) revealed a typical CT feature of COVID-19, manifested as bilateral multiple irregular areas of ground-glass opacities (GGO) and consolidation **(**Fig. 2b, c**)**. He had severe COVID-19 and was put on BiPAP ventilator. Methylprednisolone (1 mg/kg/d) and immunoglobulin (10 g/d) were intravenously administrated for 10 days. His symptoms gradually improved, body temperature returned to normal, and BiPAP ventilator was replaced by nasal cannula to maintain oxygen saturation. On Feb. 8 (day 21), he was discharged from hospital after a CT examination on Feb. 3 (day 17) showing significantly decreased lesions (Fig. 2d) and two negative oropharyngeal swab tests for SARS-CoV-2 on Feb. 4 (day 18) and Feb. 6 (day 20). He was placed on home quarantine. Five days later, he had fever again. On Feb.14, 2020 (day 27), he was admitted to the isolation ward, as he was retested positive for SARS-CoV-2 and the CT showed higher density of consolidation (Fig. 2e). The patient received oxygen support and methylprednisolone (10 mg/d) for 5 days. Within 2 days of treatment, his temperature dropped back to normal. Although the sixth CT scan showed higher density of consolidation (Fig. 2f), his symptoms disappeared completely. On March 1 (day 44), he was discharged from hospital after negative test for SARS-CoV-2 and improved absorption of inflammation on CT scan (Fig. 2g). His test for SARS-CoV-2 remained negative after 14 days of further isolation at home,.

Of note, the number of lymphocytes significantly decreased alone with aggravation and recurrence of the disease, but was recovered accompanied with improvement of respiratory symptom. LDH was elevated during the course of disease and peaked on day 13 after initial symptom onset with the lowest number of lymphocytes, and maintained at higher levels during the recurrence. In addition, serum levels of ferritin and IL2R also significantly increased after recurrence (Fig. 1), although the concentrations of these two molecules increased only a little after the initial infection (data not shown)**.**

Serological tests on Feb. 12 (day 31), 14 (day 33) and March 1 (day 44), 2020 showed lower levels of antibodies against SARS-CoV-2, respectively (Table 1). The anti-SARS-CoV-2 IgM ranged from 19.27 to 36.44 AU/ml and IgG ranged from 24.68 to 28.81 AU/ml (detail shown in supplementary method). Total exon sequencing revealed a point mutation and an insertion of 6 nucleotides of TRNT1 (tRNA nucleotidyl transferase 1) gene, encoding a CCA-adding enzyme. Mutations in this gene may be associated with B cell immunodeficiency (detail shown in supplementary method).

**Table 1:** The levels of anti-SARS-CoV-2 antibody

Detection time point (days after ill onset)	IgM (AU/ml)^a^	IgG (AU/ml)^a^
31	36.44	28.96
33	28.81	25.87
44	19.27	24.68

^a^The normal value of anti-SARS-CoV-2 antibodies is lower than 10 AU/ml

## Discussion and conclusion

This patient with recurrent pneumonia after discharging from hospital, manifested as progression of lesions on CT scan, fever, positive SARS-CoV-2 test, elevated levels of ferritin and IL2R and reappearance of lymphocytopenia. Of note, our results showed that lymphocytopenia was associated with severity of COVID-19 [5–7]. The reappearance of clinical manifestation and laboratory findings suggested a possible COVID-19 relapse. However, GGO and consolidations the typical CT imaging features of COVID-19, were also found in other viral or organizing pneumonia scans. Therefore, we could not completely rule out these possibilities in the absence of virus quantitative detection.

So far, little is known about the causes of the recurrence of SARS-CoV-2 infection after patients meet the discharge criteria. There is a possibility that the virus had not been fully removed from these patients at the first discharge, which was supported by persistently elevated levels of LDH [8]. In addition, the true positive rate of the oropharyngeal swab test for SARS-CoV-2 was only 30 to 60% [9], and the test was not sensitive enough to detect lower copies of the virus. Duration and excretion of virus in the respiratory tract of patients and performance of oropharyngeal swab also affected the results. A more sensitive technique, droplet digital PCR as a clinical trial is used for the detection of SARS-CoV-2 [10]. Although the patient did not have a contact history with other COVID-19 patients after discharge, the possibility of reinfection could not be completely ruled out during his stay in triple room of isolation ward. Insufficient production of protective antibodies might be responsible for reinfection [11] and for failure to clear the virus completely in this patient. We tracked the antibody levels of 5 patients with good recovery of the disease (detected on day 24 to day 37 after symptom onset) and found that levels of IgM and IgG were higher than that of this patient, especially IgG levels were all higher than 100 AU/ml (supplementary Table 1). Unfortunately, the patient had no serological evidence about levels of antibodies against SARS-CoV-2 during the first hospitalization. The failure of protective antibody formation might be the key reason for the possible recurrence of SARS-CoV-2 infection. Total exon sequencing demonstrated that this patient has mutations in TRNT1 gene. Mutations in this gene are associated with a rare syndrome of congenital sideroblastic anemia, B cell immunodeficiency, periodic fever, and development delay (SIFD) [12]. This seems not to be the case. Whether mutations in TRNT1 gene caused humoral immunodeficiency in this patient should be further investigated.

With more patients being discharged, similar cases have been reported in different regions. Since these patients are potentially contagious, it is paramount to extend their isolation period. In fact, policies have been implemented in many places in China to keep patients in quarantine for at least 14 days after they have met the discharge criteria. Whether this patient or patients with immunodeficiency will relapse again or become chronic infection remains unknown. Further follow-up and investigation are needed.

## Supplementary information

**Additional file 1 **Method:Real-Time reverse transcription polymerase chain reaction assay for SARS-CoV-2; total exon sequencing; Serological determination for SARS-CoV-2-specific IgM and IgG. **Supplementary Table 1**: Antibody levels of 5 patients with good recovery of COVID-19.

## Data Availability

The datasets supporting the conclusions of this article are included within the article and additional files. The datasets used during the current study are available from the corresponding author on reasonable request.

## Abbreviations

SARS-CoV-2: Severe acute respiratory syndrome corona virus 2
COVID-19: Corona virus disease − 19
BIPAP: Biphasic positive airway pressure
CT: Computed tomography
PCR: Polymerase chain reaction
