# Supplementary material for: Recurrent pneumonia in a patient with new coronavirus infection after discharge from hospital for insufficient antibody production: a case report
Source: BMC Infect Dis. 2020 Jul 11;20:500. doi: 10.1186/s12879-020-05231-z (PMC7352096; doi:10.1186/s12879-020-05231-z)
Supplement: Supplementary file 1 — Additional file 1 Method:Real-Time reverse transcription polymerase chain reaction assay for SARS-CoV-2; total exon sequencing; Serological determination for SARS-CoV-2-specific IgM and IgG. Supplementary Table 1: Antibody levels of 5 patients with good recovery of COVID-19. [file 12879_2020_5231_MOESM1_ESM.docx]

A Recurrent Case of New Corona Virus Infection after Discharge from Hospital

Xiaoxi Zhou1, Jianfeng Zhou 1, Jianpin Zhao 2

1Department of Hematology, 2Department of Respiratory, Tongji Hospital, Tongji Medical College, Huazhong University of Science and Technology, Wuhan, Hubei, China.

**Correspondence**: Jianpin Zhao Ph.D, Department of Respiratory, Tongji Hospital, Tongji Medical College, Huazhong University of Science and Technology, 1095 Jiefang Avenue, Wuhan, Hubei 430030, China; Email: [Zhaojp88@126.com](mailto:Zhaojp88@126.com).

**Method**

**Real-Time reverse transcription polymerase chain reaction assay for SARS-CoV-2**

Oropharyngeal specimens were collected for extracting SARS-CoV-2 RNA. The real-time reverse-transcriptase polymerase-chain-reaction (RT-PCR) assay method was performed to detect SARS-CoV-2. The sequences of the primers and probe were as follows:

forward primer 5′-TCAGAATGCCAATCTCCCCAAC-3′;

reverse primer 5′-AAAGGTCCACCCGATACATTGA-3′;

and the probe 5′CY5-CTAGTTACACTAGCCATCCTTACTGC-3′BHQ1.

Conditions for the amplifications were 50°C for 15 min, 95°C for 3 min, followed by 45 cycles of 95°C for 15 s and 60°C for 30 s. (The protocol of Real-time reverse-transcriptase polymerase-chain-reaction (RT-PCR) assay for SARS-CoV-2. <http://ivdc.chinacdc.cn/kyjz/202001/t20200121_211337.html>)

**Total exon sequencing**

White blood cells specimens were collected for extracting DNA. Total exon sequencing was performed by Huada gene technology company (Beijing Genomics institution).

**Serological determination for SARS-CoV-2-specific IgM and IgG**

Paramagnetic-particle chemiluminescent immunoassay (CLIA) was used to detect specific IgM and IgG against SARS-CoV-2 by iFlash-SARS-CoV-2 IgM/IgG assay kit (SHENZHEN YHLO BIOTECH CO., LTD.) and iFlash Immunoassay Analyzer (Shenzhen Yhlo Biotech., Ltd.).

**Supplementary Table 1**: Antibody levels of 5 patients with good recovery

| Cours of disease (days) | 29 | 24 | 29 | 37 | 24 |
| --- | --- | --- | --- | --- | --- |
| IgM (AU/ml) | 43.91 | 48.59 | 137.27 | 249.36 | 75.06 |
| IgG (AU/ml) | 155.34 | 118.55 | 332.77 | 170.41 | 199.33 |

*The normal value of anti-SARS-CoV-2 antibodies is lower than 10 AU/ml.
